# Oat beta-glucan as a dietary supplement for dogs

**DOI:** 10.1371/journal.pone.0201133

**Published:** 2018-07-31

**Authors:** Lívia Geraldi Ferreira, Mariangela Endrighi, Karen Guttenkunst Lisenko, Maiara Rodrigues Duarte de Oliveira, Mateus Resende Damasceno, Jelieny Aparecida Claudino, Pamella Godinho Gutierres, Ana Paula Peconick, Flávia Maria de Oliveira Borges Saad, Márcio Gilberto Zangeronimo

**Affiliations:** 1 Veterinary Medicine Department, Federal University of Lavras, Lavras, Minas Gerais, Brazil; 2 Animal Science Department, Federal University of Lavras, Lavras, Minas Gerais, Brazil; University of Illinois, UNITED STATES

## Abstract

The aim of this study was to evaluate the effects of oat beta-glucan supplementation on metabolic, physiological, immunological and nutritional variables in adult dogs. Fourteen dogs were fed a control diet or a diet supplemented with 1% beta-glucan during 71 days. Serum concentrations of glucose, total cholesterol and lipoprotein fractions, as well as plasma concentrations of peptide YY and ghrelin, were determined. In addition, coefficient of total tract apparent macronutrient digestibility (CTTAD), food intake and fecal output, score, and pH were evaluated. For evaluation of immunological variables, serum concentrations of interleukin-4 and interferon gamma were determined on days 0, 57 and 71, which corresponded to blood collection prior to beta-glucan supplementation, and at 7 and 14 days post first and second dose of vaccination (Pneumodog^®^, Merial, Campinas, Brazil), respectively. Animals fed the supplemented diet showed (P<0.05) lower serum concentrations of total cholesterol and low and very low density lipoproteins, lower coefficients of total tract apparent digestibility of dry matter, organic matter, mineral matter and ether extract, higher fecal output and lower fecal consistency, as well as a tendency (P = 0.07) of decreasing the coefficient of total tract apparent digestibility of crude protein. Moreover, the supplemented diet resulted in lower number of red blood cells, hematocrit percentage and hemoglobin concentration 21 days post-vaccination as well as lower serum concentration of interleukin-4 seven days post-vaccination (P<0.05). It is concluded that oat beta-glucan extract can be used as a dietary supplement for dogs at a dose of 10 g/kg of food, being effective in reducing blood concentrations of total cholesterol, LDL-c and VLDL-c as well as CTTAD of nutrients, demonstrating a potential to be used in the feeding of obese animals. In addition, by reducing the predominance of a Th2 response, oat beta-glucan can positively modulate the vaccine response of animals.

## Introduction

Beta-glucans represent one of the major structural components of the cell wall of fungi, yeasts, and cereals, as well as some bacteria and algae [1]. In cereals, particularly, beta-glucans are presented as linear polysaccharides, in which glucose monomers are bound by β-(1,3) and β-(1,4) linkages and are mainly found in barley, oats and wheat [2–4]. This structural organization confers water solubility to beta-glucans, which are therefore classified as soluble fibers [5].

For this reason, several studies with humans [6, 7] and mice [8, 9] have evaluated the ability of beta-(1,3)-(1,4)-glucan to positively influence physiological and metabolic processes in the body such as satiety stimulation, reduction of blood glucose and cholesterol concentrations and body weight reduction. These effects may significantly contribute to the prevention and treatment of disorders, such as obesity [3], the most commonly observed nutritional problem in dogs [10]. Moreover, beta-glucan from oats is a natural product that may have a positive tag appeal to pet owners. However, the effects of diet supplementation with purified preparations of this kind of beta-glucan on dogs have not yet been proven.

In addition, dogs are often subjected to a variety of stress factors, such as poor housing conditions, harsh training sessions and uncontrollable or unpredictable social environments that may interfere with hematological and immunological parameters[11]. Obesity is also related to a decrease in immune function, with obese dogs showing less resistance to the development of infections [10]. Thus, the use of compounds like beta-glucans that are able of allying metabolic, physiological and immunological benefits, present great potential for being further explored in animal nutrition, especially in companion animal nutrition.

Few studies have demonstrated the performance of beta-glucans as immunomodulatory agents [12–14]. Such ability is associated with the recognition of beta-glucan as a pathogen associated molecular pattern (PAMP) by different cells of the immune system [15]. This recognition results in the activation of these cells and subsequent cytokine production [7]. However, most of these studies evaluated the effects of beta-glucans extracted from fungi and yeasts, which present the glucose monomers bound by beta-(1,3) and beta-(1,6) linkages; those are structurally different from cereal beta-glucans [1]. Hence, further studies on cereal beta-glucans are necessary to determine the nature of the immunological effects of these compounds.

Therefore, the aim of this study was to evaluate the effects of dietary supplementation with oat beta-glucan extract on physiological, metabolic, immunological and nutritional parameters in adult dogs.

## Materials and methods

### Animals, facilities and experimental design

The experiment was conducted at the Center for Studies on Companion Animal Nutrition (CENAC) in the Department of Animal Sciences at the Federal University of Lavras, located in Lavras, Minas Gerais, Brazil. The entire experimental procedure was approved by the institution's Ethics Committee on Animal Use (protocol no. 005/2015).

Fourteen adult Beagles belonging to the CENAC were used. The animals aged 6.14 ± 3.13 years, weighing 16.2 ± 3.2 kg, and with body score condition of 4.00 ± 0.78 [16] were kept in individual kennels of 4.8 m^2^ (1.2 m wide x 4.0 m long) with an indoor area (a covered area with 1.2 m wide x 2.0 m long) and an outdoor area (solarium with 1.2 m wide x 2.0 m long). Each kennel was equipped with troughs and nipple drinkers. During the experiment, the maximum and minimum temperatures were 30.3 ± 2.8 °C and 18.1 ± 2.8 °C, respectively, and the values of maximum and minimum humidity were 78.4 ± 6 and 44.2 ± 4.2%, respectively. The animals, all in perfect health conditions, were distributed in a randomized complete block design (weight × age) with two treatments (with and without beta-glucan supplementation) and seven replicates of one animal each. The experimental period was 71 days.

### Experimental diets

Experimental diets consisted of a commercial dry food (Tables 1 and 2) formulated to meet the nutrient requirements of adult dogs in maintenance [17], with or without supplementation of beta-glucan extracted from oats. The supplementation was done with a commercial product with 70% purity as is basis (B-CAN 70^®^, Embrafarma, São Paulo, Brazil) (Table 2). According to the product´s manufacturer, B-CAN 70^®^ is composed of 70% beta-glucan, 12.1% non-fibrous carbohydrates, 3.43% protein, 1.42% fat and 154mg of sodium. The product is concentrated through an extraction process with water and ethanol followed by drying.

**Table 1:** Chemical composition (minimum and maximum) of the commercial diet used^a^, according to the label, as is basis.

Item	Inclusion (g/kg)
**Moisture (max.)**	100.00
**Crude protein (min.)**	220.00
**Ether extract (min.)**	100.00
**Crude fiber (max.)**	30.00
**Mineral matter (max.)**	90.00
**Calcium (min., max.)**	10.00–20.00
**Phosphorus (min., max.)**	8.00–12.00
**Potassium (min.)**	5.00
**Sodium (min.)**	2.00
**Lysine (min.)**	8.00
**Omega 3 (min.)**	3.00
**Omega 6 (min.)**	24.00
**Sodium hexametaphosphate (min.)**	3.00
**Organic zinc (min.)**	0.05

^a^ Dry, extruded food for the feeding of adult dogs (Three Dogs, Hercosul, Ivoti, Rio Grande do Sul, Brazil).

Ingredient composition: mixture of fresh meat (beef and pork) (min. 5%), bovine meat and bone meal, poultry by-product meal, poultry and pork liver hydrolyzate, brewers rice, grounded whole grain corn, flaxseed, stabilized animal fat, wheat bran, sodium chloride, sodium hexametaphosphate, potassium chloride, choline chloride, L-lysine, yucca extract, probiotic, antioxidants (BHA/BHT), vitamins (A, B1, B12, B2, B6, D3, E, K3, folic acid, pantothenic acid, biotin and niacin) and minerals (zinc proteinate, calcium iodate, sodium selenite, copper sulfate, manganese sulfate, zinc sulfate and ferrous sulfate).

**Table 2:** Chemical composition of the commercial diet supplemented or not with beta-glucan and of the beta-glucan commercial preparation used.

Variables	Control diet	Supplemented diet	Beta-glucan commercial preparation
**Dry matter (DM), g/kg**	934.4	934.5	942.6
**Organic Matter, g/kg in DM basis**	970.4	970.2	948.0
**Crude Protein, g/kg in DM basis**	250.3	248.1	35.8
**Acid hydrolyzed ether Extract, g/kg in DM basis**	134.3	133.1	15.9
**Crude fiber, g/kg in DM basis**	32.1	33.4	165.0
**Neutral Detergent Fiber, g/kg in DM basis**	144.5	150.5	742.6
**Non-nitrogenous extract, g/kg in DM basis**	492.3	494.7	731.3
**Metabolizable energy, kcal/kg in DM basis**^**1**^	3,665	3,653	2,489

^1^ Estimated values according to ABINPET [18]

Animals were fed at 9.00 hours, after the cleaning procedures of the kennels were done. The amount of energy required by each animal, in kcal, was calculated by the formula 130 × body weight^0.75^, which is recommended for adult dogs in maintenance [17]. Then, the amount of food supplied, in grams, was calculated on the basis of the metabolizable energy (ME) value of the food used. The ME of food was calculated by the following formulas [18]:

GE=(5.7×CPingrams)+(9.4×AHEEingrams)+[4.1×(NNEingrams+CFingrams)]

where GE is the gross energy; AHEE is the acid hydrolyzed ether extract; NNE is the non-nitrogenous extract and CF is the crude fiber.

DE=(GE×CDE)÷100

where DE is the digestible energy and CDE is coefficient of digestible energy calculated by the formula CDE = 91.2 –(1.43 x % CF, in a dry matter basis)

ME=DE–(1.04×CPingrams)

Approximately 5 minutes before feeding, the animals of the test group received beta-glucan dissolved in 10 mL of water through a syringe. The time between feeding the first animal and the last animal was less than 20 minutes. The amount of beta-glucan was calculated for each animal according to the amount of food supplied (10 g of beta-glucan/kg of food). The animals of the control group received only water through the same procedure described for the animals of the test group.

Animals were weekly weighed to calculate the amount of food and beta-glucan. Water was given *ad libitum*.

### Determination of metabolic and physiological parameters

**Fig 1:**
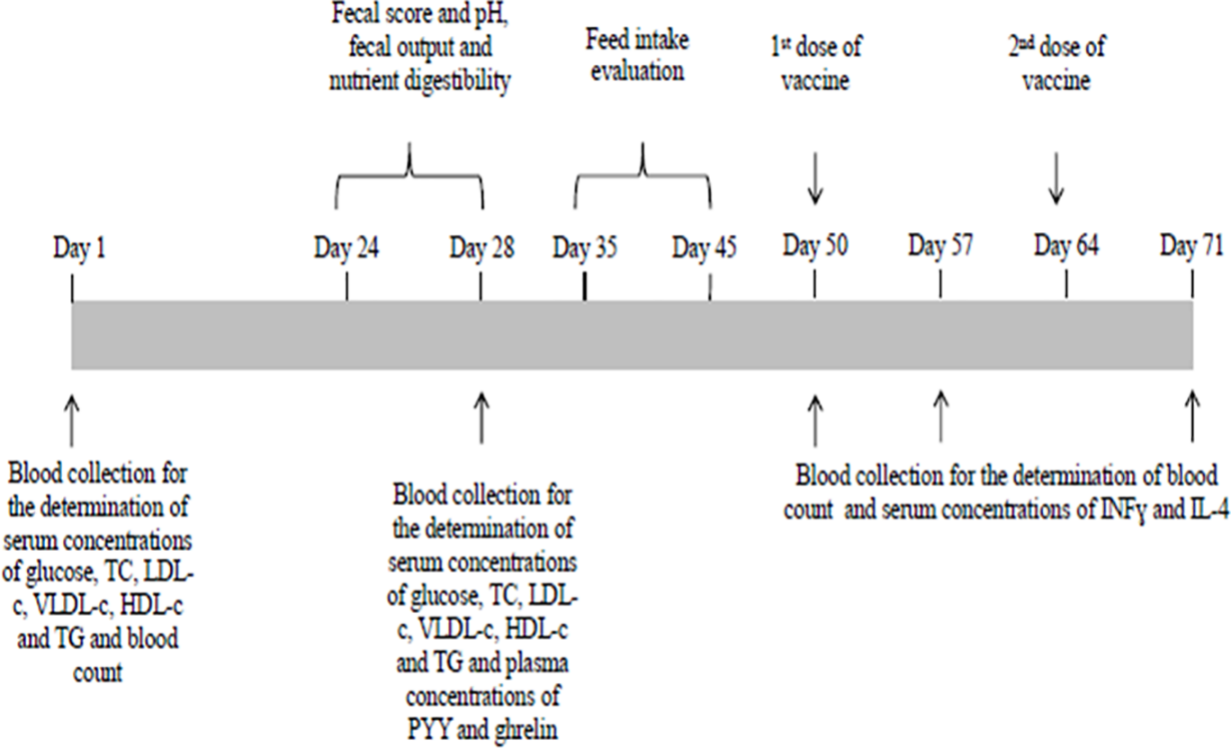
Timeline of experimental period and sample collection. TC: total cholesterol, LDL-c: cholesterol in low density lipoprotein, VLDL-c: cholesterol in very low density lipoprotein, HDL-c: cholesterol in high density lipoprotein, TG: triacylglycerols, INFɣ: interferon ɣ, IL-4: interleukin-4.

The flowchart of the experimental procedure is presented in Fig 1. On the first day of the study, 10 mL of blood were collected from each animal in sterile, plastic, vacuum test tubes without anticoagulant (Labor Import, Osasco, São Paulo, Brazil) through puncture of the jugular vein to determine the serum concentrations of glucose (glucose oxidase method; Glicose Liquiform ref. 133–2, Labtest, Lagoa Santa, Minas Gerais, Brazil), total cholesterol (enzymatic colorimetric method; Colesterol Liquiform ref. 76–2, Labtest, Lagoa Santa, Minas Gerais Brazil), cholesterol in high-density lipoprotein (HDL-c) (selective surfactant method; HDL ref. 145–1, Labtest, Lagoa Santa, Minas Gerais, Brazil), low density lipoprotein (LDL-c) (calculation; LDL-c = (triacylglycerols/5 + HDL-c)–total cholesterol) and very low density lipoprotein (VLDL-c) (calculation; VLDL-c = triacylglycerols/5) and triacylglycerols (enzymatic colorimetric method), using a automatic analyzer (Labmax 240, Labtest, Lagoa Santa, Minas Gerais, Brazil). Blood samples were taken from animals fasted for 12 hours and immediately sent to a commercial clinical laboratory. Blood samples were also taken after 60 and 120 minutes of feeding for the determination of serum glucose concentrations.

On day 28 of experiment, blood samples were collected under the same conditions previously described, before and after 60 and 120 minutes of feeding for the determination of the same blood variables afore mentioned [19]. In addition, blood samples were also collected in sterile, plastic, vacuum test tubes containing ethylenediaminetetra-acetic acid (EDTA) (Labor Import, Osasco, São Paulo, Brazil) for peptide YY (PYY) and ghrelin quantification. These blood samples were immediately transferred to test tubes containing aprotinin in the concentration of 0.6 trypsin inhibitor units (TIU)/mL, according to the manufacturer's recommendations. Thereafter, the tubes were centrifuged at 1600 g for 15 minutes at 4 °C and the plasma was transferred to microtubes, which were stored in a freezer at -80 °C until analysis. Plasma concentrations of PYY and ghrelin were determined by competitive enzyme immunoassays (ELISA) using commercial kits (EIA KIT EK-059-03 PYY for rat, mouse, porcine and canine; and EIA KIT EK-031-50 Ghrelin for canine–extraction free; Phoenix Pharmaceuticals, Burlingame, USA).

### Fecal characteristics, nutrient digestibility and food intake

Fecal output, score and pH were evaluated between days 24 and 28 of the experiment. Fecal score was determined according to the following system [20]: 1 –hard, dry pellets; small, hard mass; 2 –hard, formed, dry stool; remains firm and soft; 3 –soft, formed, and moist stool; 4 –soft, unformed stool; assumes shape of container; 5 –watery; liquid that can be poured. Fecal pH was measured in fresh feces, collected within 15 minutes of defecation, using a digital pH meter (model Q400A, Quimis, São Paulo, Brazil) [21, 22]. The electrode was inserted in three distinct points of the sample and the average was considered in the analyzes.

After fecal score evaluation, feces were collected in plastic bags, closed and stored at -20 °C. At the end of the collection period, fecal samples from each animal were thawed at room temperature for 12 hours, weighed and homogenized. They were then placed in aluminum trays, weighed, and dried in a forced-air oven (MA035/5, Marconi^®^, Piracicaba, São Paulo, Brazil) at 65 °C for 72 hours. After reaching the room temperature, fecal samples were weighed and grounded in a Thomas-Wiley hammer mill, using a 1.0 mm screen. The samples were stored in plastic containers at room temperature for further analysis.

Diets and fecal samples were analyzed for dry matter (DM) (method 934.01), crude protein (CP) (method 954.01) and mineral matter (MM) (method 942.05) according to methodologies described by the Association of Official Analytical Chemists [18]. The lipid content was determined by acid hydrolysis followed by ether extraction (acid hrydolyzed ether extract–AHEE) according to methodologies described by the AACC [23] and Budde [24]. The neutral detergent fiber content of the food was determined according to methodologies described by AOAC [25].

The food intake evaluation by the satiety test was performed between days 35 and 45 of the experiment. Beta-glucan mixed with water or water alone was administered to the animals following the same procedures previously described. Subsequently, food was provided *ad libitum* for 30 minutes and leftovers were weighed for food intake determination. Food intake data were evaluated as grams of food consumed per kg of body weight.

### Determination of immunological parameters

On the first day of the experiment, blood samples were collected in sterile, plastic, vacumm tubes containing EDTA (Labor Import, Osasco, São Paulo, Brazil) for blood count evaluation. On day 50, animals received the first dose of the vaccine (Pneumodog®, Merial, Campinas, São Paulo, Brazil) and seven days later (day 57), blood samples were collected for evaluation of complete blood count and serum interferon gamma (INFɣ) and interleukin-4 (IL-4) concentrations. The dogs were completely naive to the vaccine. On day 64, animals received the second dose of the vaccine following the vaccine protocol established by the manufacturer, and, seven days later (day 71; 21 days after the first dose of the vaccine and seven days after the second dose of the vaccine) blood samples were collected again for evaluation of the same variables described for day 57. Both vaccination and blood sample collection were performed on fasted animals. Part of the blood samples was collected in sterile, plastic, vacuum test tubes (Labor Import, Osasco, São Paulo, Brazil) containing EDTA and immediately sent to the commercial clinical laboratory for the complete blood count, which was performed using an automated hematology analyzer (pocH– 100iV, Sysmex, São Paulo, São Paulo, Brazil). The other part was collected in sterile, plastic, vacuum test tubes without anticoagulant (Labor Import, Osasco, São Paulo, Brazil) and centrifuged at 1000 g for 15 minutes at 4 °C. The serum was then pipetted into microtubes, which were stored in a freezer at -80 °C until the analyses were performed. Serum concentrations of INFɣ and IL-4 were determined by sandwich ELISA using commercial kits (CAIF00 Canine INF-gamma quantikine ELISA KIT; DY754 Canine IL-4 Duoset; R & D Systems, Minneapolis, USA), following the manufacturer's specifications.

### Calculations and statistical analyses

The coefficient of total tract apparent digestibility (CTTAD) of DM was calculated using the formula: CTTAD_DM_ (%) = ((a-b)/a) x 100, where *a* = food intake on DM basis and *b* = fecal output on DM basis. The CTTAD of other nutrients was calculated using the formula: CTTAD_nutrient_ (%) = (((a x b–c x d)) / (a x b)) x 100, where *a* = food intake on DM basis, *b* = nutrient percentage in feed, *c* = fecal output on DM basis and *d* = nutrient percentage in feces.

Data were submitted to the normality test by the Shapiro-Wilk test, homoscedasticity of variances by the Breusch Pagan test and independence of errors by the Durbin-Watson test. When all conditions were met (P>0.05), analysis of variance (ANOVA) was performed and the means were compared by the F test. Otherwise, nonparametric analysis was performed and means were compared by the Friedman test. The serum concentrations of glucose, total cholesterol, HDL-c, LDL-c, VLDL-c and triacylglycerols, as well as blood count parameters, were submitted to covariance analysis and the values of each variable determined before the beginning of the experiment were assumed to be covariates. For analyzes that were evaluated in time, repeated-measures split-plot analysis of variance were used. All statistical analyses were performed using the Action 3.1 statistical package.

## Results

### Body weight, food intake and fecal characteristics

**Fig 2:**
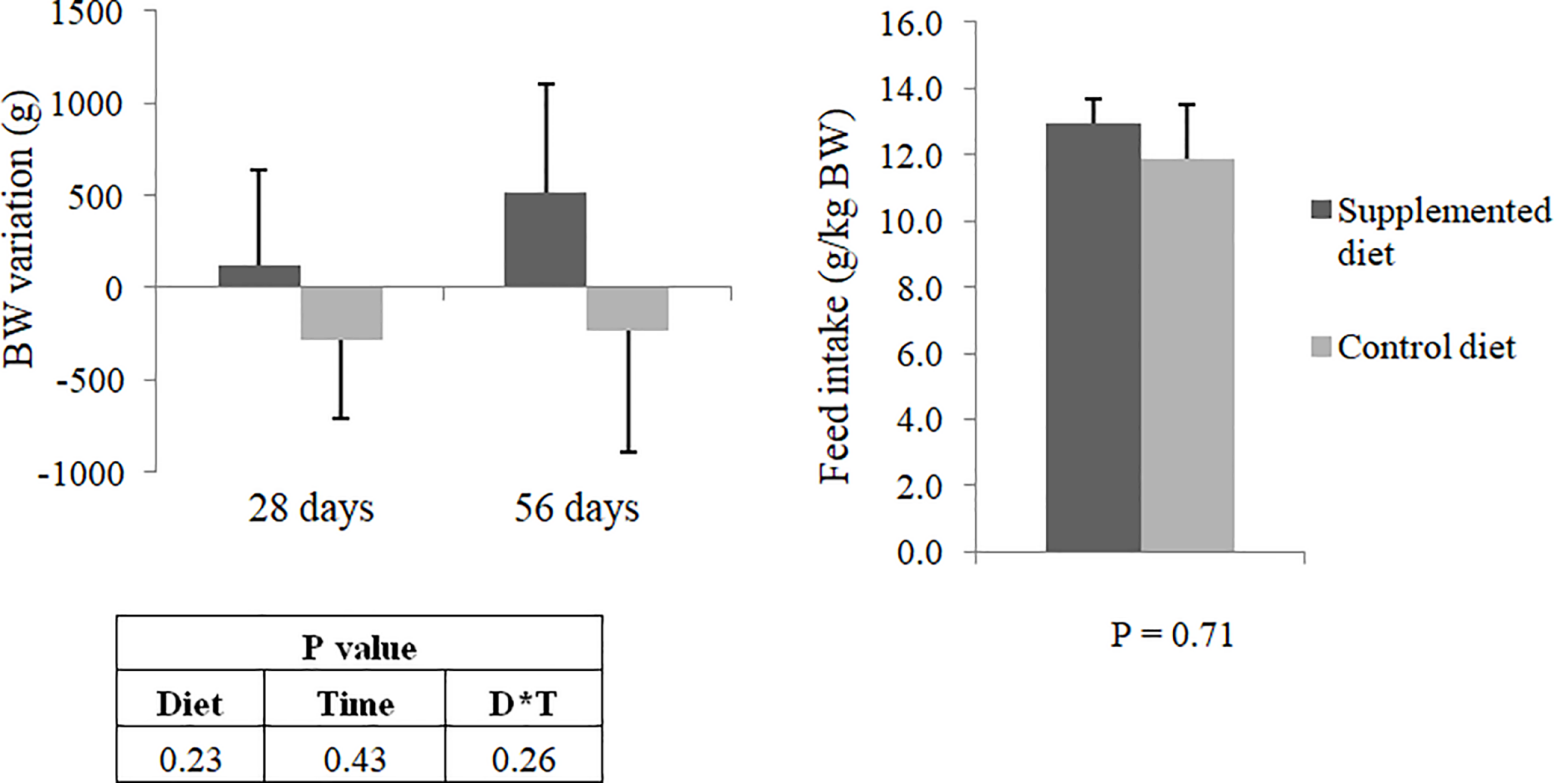
(a) Average body weight variation (g) at 28 and 56 days of the study and (b) Average food intake (g/kg BW) performed between days 35 and 45 of dogs fed the control or supplemented diet with beta-glucan. BW: body weight. No significant differences by the Friedman test (P>0.05).

**Fig 3:**
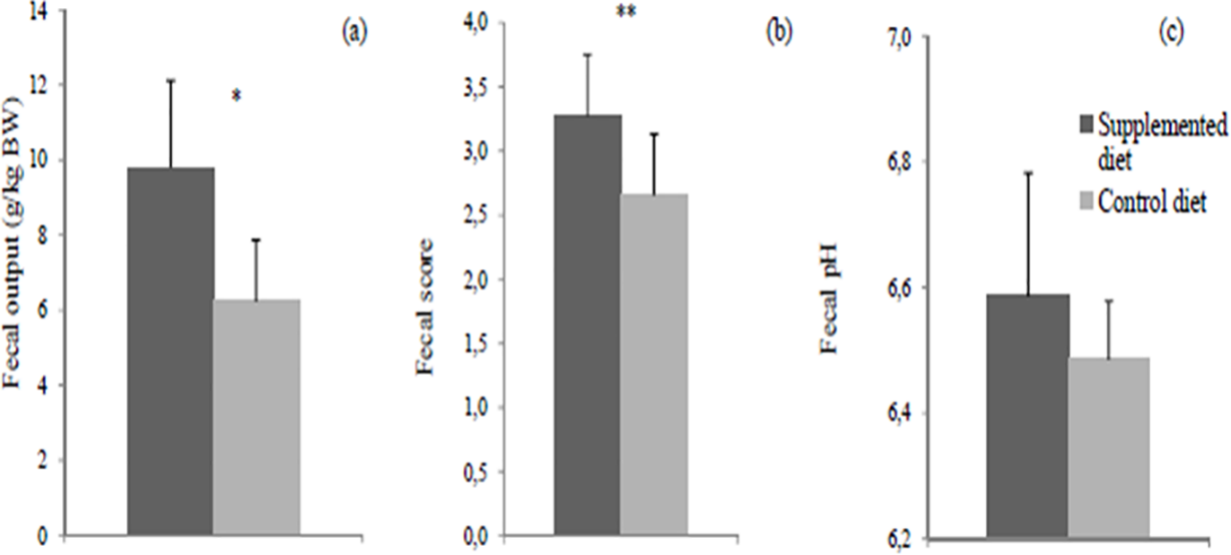
Fecal characteristics of dogs fed the control or supplemented diet with beta-glucan, evaluated between days 24 and 28 of the study. (a) Fecal output (g/kg BW). (b) Fecal score determined according to the following system [20]: 1 –hard, dry pellets; small, hard mass; 2 –hard, formed, dry stool; remains firm and soft; 3 –soft, formed, and moist stool; 4 –soft, unformed stool; assumes shape of container; 5 –watery; liquid that can be poured. (c) Fecal pH. BW: body weight. *Means differ by the F test (P<0.05). **Means differ by the Friedman test (P<0.05).

The average daily food intake during the experimental period was 311 ± 44 g for control group and 315 ± 50 g for supplemented group. The average BG intake by the test group was 3.15 ± 0.50 g. Average body weight variation of animals throughout the study and average food intake during the satiety test were not influenced (P>0.05) by dietary beta-glucan (Fig 2). However, supplemented diet increased (P<0.05) fecal output and decreased (P<0.05) fecal consistency (Fig 3). There was no effect (P>0.05) on fecal pH.

### Nutrient digestibility

Beta-glucan supplementation reduced (P<0.05) the CTTAD of DM, organic matter (OM), MM and AHEE when compared with the control diet. A tendency (P = 0.07) of decreasing the CTTAD of CP was also observed (Table 3).

**Table 3:** Coefficient of total tract apparent digestibility of nutrients (± standard error mean) of the control or supplemented diet with beta-glucan.

Coefficient of total tract apparent digestibility (%)	Control diet	Supplemented diet	P-value
**Dry matter**	83.0 ± 1.4	78.4 ± 0.9*	0.05
**Organic matter**	86.4 ± 1.0	82.6 ± 0.8*	0.04
**Mineral matter**	41.7 ± 1.3	24.1 ± 0.8*	0.04
**Crude protein**	85.4 ± 0.6	81.7 ± 1.0	0.07
**Acid hydrolyzed ether extract**	93.1 ± 4.6	88.9 ± 3.2*	0.01

* Mean values were significantly different from those of the control group (P<0.05).

### Blood parameters

**Fig 4:**
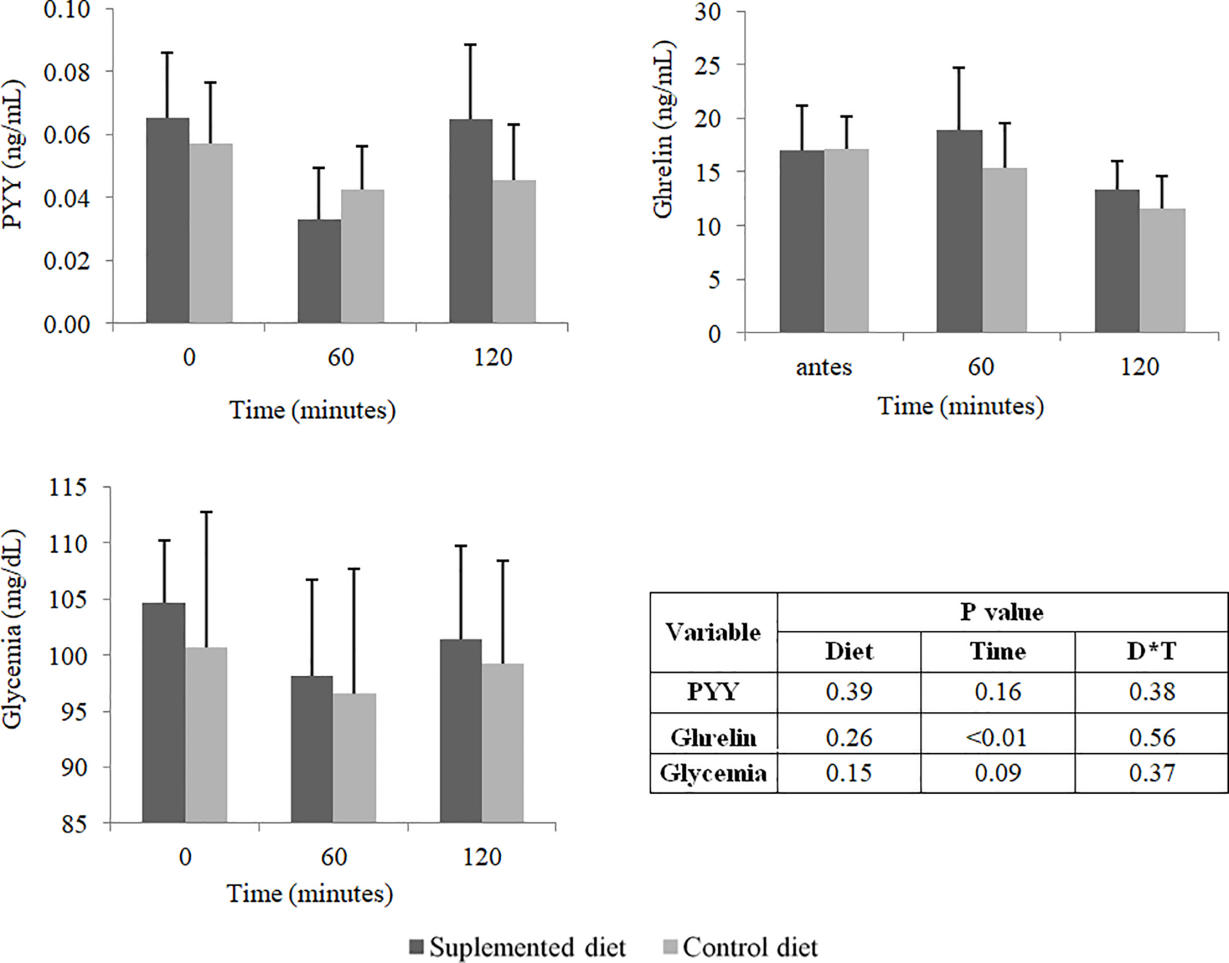
(a) Plasma concentrations of PYY (ng/mL) and (b) ghrelin (ng/mL) and (c) glycemia (mg/mL) of dogs fed the control or supplemented diet with beta-glucan. Values determined before feeding (time 0), 60 minutes after feeding (time 60) and 120 minutes after feeding (time 120). No significant differences by the Friedman test (P<0.05).

**Fig 5:**
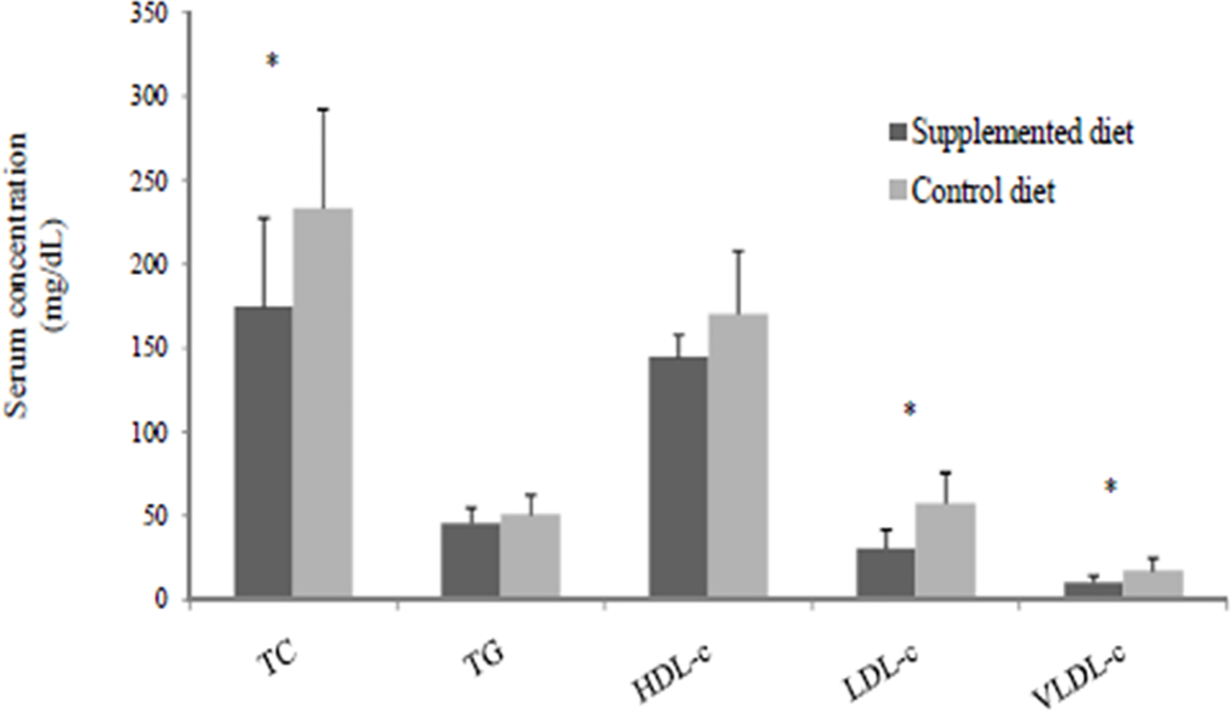
Serum lipid fractions of fasted dogs fed the control or supplemented diet with beta-glucan for 28 days. TC: total cholesterol; TG: triacylglycerols; HDL-c: cholesterol in high density lipoprotein; LDL-c: cholesterol in low density lipoprotein; VLDL-c: cholesterol in very low density lipoprotein.* Means differ by the F test (P<0.05).

There was no effect (P>0.05) of the dietary beta-glucan on glycemia and plasma concentrations of PYY and ghrelin at 60 and 120 minutes after the supplementation (Fig 4). However, supplemented diet reduced (P<0.05) serum concentrations of total cholesterol, LDL-c and VLDL-c at day 28 of the experiment (Fig 5).

Beta-glucan supplementation did not influence (P>0.05) blood count before the vaccination of animals (Tables 4 and 5). However, increased red blood cell number, hemoglobin concentration, and hematocrit percentage (P<0.05) were observed seven days after the second dose of the vaccine in animals receiving beta-glucan supplementation. There was no effect (P>0.05) on other parameters of the blood count.

**Table 4:** Blood count (± standard error mean) of dogs fed the control or supplemented diet with beta-glucan evaluated before and after vaccination^a^.

Variable	Time of blood collection	Control diet	Supplemented diet	P value		
				Diet	Time	D*T
**Red blood cells (millions/mm**^**3**^**)**	**Before vaccine**	7.24 ± 0.29	7.61 ± 0.23	0.17	0.10	0.16
	**7 days after the 1**^**st**^ **dose**	7.43 ± 0.25	7.81 ± 0.19			
	**7 days after the 2**^**nd**^ **dose**	7.22 ± 0.29	7.81 ± 0.22*			
**Hemoglobin (g/dL)**	**Before vaccine**	16.73 ± 0.79	17.67 ± 0.65	0.13	0.57	0.57
	**7 days after the 1**^**st**^ **dose**	16.90 ± 0.62	17.74 ± 0.38			
	**7 days after the 2**^**nd**^ **dose**	16.41 ± 0.73	17.73 ± 0.46*			
**Hematocrit (%)**	**Before vaccine**	47.26 ± 2.06	49.87 ± 1.70	0.13	0.76	0.33
	**7 days after the 1**^**st**^ **dose**	47.61 ± 1.78	49.81 ± 1.13			
	**7 days after the 2**^**nd**^ **dose**	46.37 ± 2.08	50.23 ± 1.14*			
**Mean corpuscular volume (fL)**	**Before vaccine**	65.17 ± 0.59	65.49 ± 0.93	0.81	0.11	0.58
	**7 days after the 1**^**st**^ **dose**	64.00 ± 0.67	63.83 ± 0.90			
	**7 days after the 2**^**nd**^ **dose**	64.11 ± 0.63	64.39 ± 0.93			
**Mean corpuscular hemoglobin (pg)**	**Before vaccine**	23.01 ± 0.22	23.14 ± 0.41	0.82	0.12	0.89
	**7 days after the 1**^**st**^ **dose**	22.69 ± 0.19	22.71 ± 0.36			
	**7 days after the 2**^**nd**^ **dose**	22.67 ± 0.17	22.69 ± 0.36			
**Mean corpuscular hemoglobin concentration (%)**	**Before vaccine**	35.33 ± 0.20	35.36 ± 0.15	0.95	0.31	0.70
	**7 days after the 1**^**st**^ **dose**	35.46 ± 0.17	35.59 ± 0.13			
	**7 days after the 2**^**nd**^ **dose**	35.36 ± 0.14	35.23 ± 0.19			
**Red cell distribution width (%)**	**Before vaccine**	12.17 ± 0.34	13.39 ± 0.24	0.12	0.09	0.47
	**7 days after the 1**^**st**^ **dose**	11.66 ± 0.32	12.30 ± 0.46			
	**7 days after the 2**^**nd**^ **dose**	11.50 ± 0.38	12.59 ± 0.55			
**Platelets (thousands/mm**^**3**^**)**	**Before vaccine**	312.3 ± 19.23	335.4 ± 24.95	0.89	0.10	0.74
	**7 days after the 1**^**st**^ **dose**	369.4 ± 29.99	361.7 ± 27.79			
	**7 days after the 2**^**nd**^ **dose**	359.9 ± 51.37	362.4 ± 30.53			

^a^ Pneumodog®, which is indicated for the prevention of respiratory diseases in dogs caused by *Bordetella bronchiseptica* and *Parainfluenza* virus type 2, administered in two doses with a 14-day interval.

* Mean values were significantly different from those of the control group (P<0.05).

**Table 5:** Leukogram (± standard error mean) of dogs fed the control or supplemented diet with beta-glucan evaluated before and after vaccination^a^.

Variable	Time of blood collection	Control diet	Supplemented diet	P value		
				diet	time	D*T
**Total leukocytes (thousands/mm**^**3**^**)**	**Before vaccine**	8.51 ± 0.30	8.20 ± 0.58	0.72	0.09	0.49
	**7 days after the 1**^**st**^ **dose**	7.81 ± 0.43	8.53 ± 0.67			
	**7 days after the 2**^**nd**^ **dose**	9.14 ± 0.96	9.27 ± 0.80			
**Segmented (%)**	**Before vaccine**	69.14 ± 3.97	70.43 ± 2.80	0.40	0.16	0.95
	**7 days after the 1**^**st**^ **dose**	70.57 ± 3.72	72.71 ± 2.35			
	**7 days after the 2**^**nd**^ **dose**	66.43 ± 4.36	69.00 ± 3.10			
**Lymphocytes (%)**	**Before vaccine**	25.86 ± 3.60	24.29 ± 3.36	0.28	0.27	0.91
	**7 days after the 1**^**st**^ **dose**	25.29 ± 3.20	22.43 ± 2.70			
	**7 days after the 2**^**nd**^ **dose**	27.86 ± 4.04	25.14 ± 3.40			
**Monocytes (%)**	**Before vaccine**	2.14 ± 0.46	2.00 ± 0.22	0.91*	0.56*	-
	**7 days after the 1**^**st**^ **dose**	2.00 ± 0.53	2.29 ± 0.68			
	**7 days after the 2**^**nd**^ **dose**	1.71 ± 0.18	1.71 ± 0.30			
**Eosinophils (%)**	**Before vaccine**	2.57 ± 0.37	3.29 ± 0.78	0.91*	0.64*	-
	**7 days after the 1**^**st**^ **dose**	1.57 ± 0.95	2.29 ± 0.68			
	**7 days after the 2**^**nd**^ **dose**	2.43 ± 0.72	3.00 ± 0.50			

^a^ Pneumodog®, which is indicated for the prevention of respiratory diseases in dogs caused by *Bordetella bronchiseptica* and *Parainfluenza* virus type 2, administered in two doses with a 14-day interval.

* No significance by Friedman test (P>0.05).

**Fig 6:**
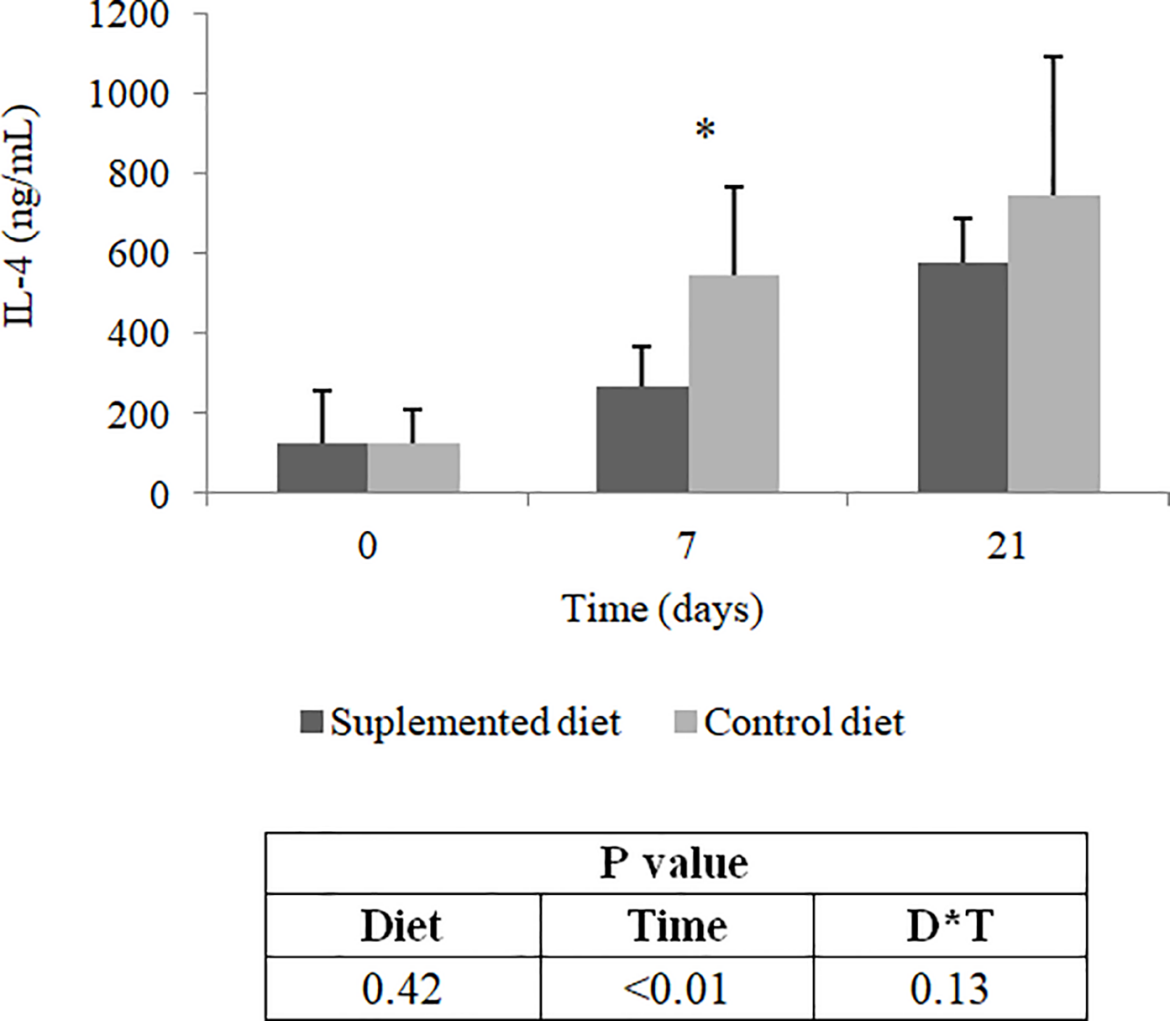
Interleukin-4 serum concentrations of dogs fed control or supplemented diet with beta-glucan. Values determined before vaccination (time 0), seven days after the first dose of vaccine (time 7) and 21 days after the first dose of vaccine (time 21). IL-4: interleukin-4. * Means differ by the Friedman test (P<0.05).

In addition, dietary beta-glucan reduced (P<0.05) serum concentrations of IL-4 seven days after the first dose of the vaccine (Fig 6). This effect was not observed (P>0.05) after the second dose of the vaccine. No detectable amounts of INFɣ were found in serum samples of experimental animals by the methodology used.

## Discussion

To our knowledge, this is the first study to evaluate the effects of the use of beta-glucan extracted from oats as a dietary supplement for dogs. The dose tested in this study was close to those used for other types of functional fibers in dogs such as mannanoligosaccharide and fructoligosaccharide [26].

The lower CTTAD of DM, OM, MM and EE was expected as beta-glucans are considered soluble fibers [5], increasing viscosity of the gastrointestinal content and hindering the action of digestive enzymes [27]. In addition, the decreasing tendency observed in the CTTAD of CP can also be attributed to the afore mentioned increased viscosity and we believe that higher doses of beta-glucan could confirm this result. In piglets, the higher level of β- glucans (18.0 vs. 15.4 g/kg) in the diet decreased nutrient digestibility [28]. However, in dogs, the effect of beta-glucan supplementation on nutrient digestibility has not been studied. The use of a commercial yeast cell wall preparation, a product that has beta-(1,3),(1,6)-glucan in its composition, as a dietary supplement for dogs in amounts ranging from 0% to 0.65%, led to a cubic response of the CTTAD of DM, OM, CP and EE [29]. The lowest values observed in that study were obtained with 0.25% supplementation. Additionally, the consumption of diets with increasing levels of soluble non-starch polysaccharides (NSPs) (11, 16 and 20g/kg), by substituting wheat for barley, an ingredient rich in beta-(1,3),(1,4)-glucan, reduced starch, fat, protein and energy digestibilities in dogs [21]. However, in this same study, the addition of an enzyme complex composed of xylanase, beta-glucanase and amylase to the diets reversed these effects. This observation was attributed to the action of these enzymes on soluble arabinoxylans and beta-glucans forming smaller polymers with reduced capacity to increase the viscosity of digesta in the small intestine, which allows digestive enzymes to have greater access to dietary components [30]. As studies that evaluated the influence of diets supplemented with beta-glucans on the CTTAD in dogs are scarce, we suggest this type of analysis in future studies with beta-glucans in this species.

The reduction in nutrient digestibility resulted in higher fecal production with feces of lower consistency when compared with the control group. Such characteristics may be considered undesirable, especially in the case of dogs living close to their owners [31]. However, despite the lower fecal consistency, fecal score values ​​observed in this study were still within the range considered normal and desirable [20].

Lower fecal consistency was also observed as levels of soluble NSPs increased in the diet of dogs [21]. This observation was due to the fact that the viscous nature of soluble NSPs causes an increase in water retention as chyme passes through the gastrointestinal tract (GIT) [32]. In addition, fermentation products, as well as carbohydrate molecules that escape the fermentative processes, exert an osmotic effect, attracting more water to the intestinal lumen, which consequently reduces fecal consistency [33].

Although a reduction in nutrient digestibility was verified, there were no differences in body weight of animals during the experimental period. Differently, dietary supplementation with barley beta-glucan [9] or oat beta-glucan [34] resulted in significant weight losses at doses of 2% and 4% (barley) or 10% (oats) in mice and rats. However, these doses are greater than the one used in the present study. Thus, there is a need for studies with higher levels of beta-glucan supplementation in dogs.

In some species, such as humans, mice, swine and broilers, studies have shown that diet supplementation with cereal beta-glucan reduces glucose, cholesterol and lipid absorption in the GIT, contributing to reductions in glycemia [6, 35] and cholesterolemia [6, 8, 36]. These results are attributed to the increased viscosity of luminal content and the reduced action of digestive enzymes on food substrate. In fact, in this study, lower lipid digestibility was observed in animals that received the diet supplemented with beta-glucan. This result partly explains the lower serum concentrations of VLDL-c, LDL-c and total cholesterol observed in animals. In addition, the increased viscosity of intestinal contents also inhibits the resorption of bile salts in the distal portion of the ileum, increasing fecal cholesterol excretion [37, 38]. Similarly, the consumption of a barley-based diet by broilers resulted in lower concentrations of total cholesterol and LDL-c compared with consumption of a soybean meal and maize-based diet [36].

In swine, dietary supplementation with different levels of oat beta-glucan (0%, 3% and 6%), which are greater than the dose used in our study, resulted in lower glucose concentration in the portal circulation after food consumption [35]. Likewise, beverages supplemented with 5 grams of oat beta-glucan in diets for humans resulted in lower values of postprandial glycemia [6]. However, in the present study, glycemia of animals was not influenced by dietary supplementation with beta-glucan, indicating that the dose used may not have been sufficient to influence glucose digestion and absorption in animals.

Regarding the influence of beta-glucan on appetite regulation, no differences in PYY and ghrelin plasma concentrations were observed among experimental groups, which partly explains the results observed in the food intake evaluation. It is known that PYY has an anorexigenic effect [39], while ghrelin has an orexigenic effect [40]. Human studies have shown that consumption of oat beta-glucan results in an increase in PYY plasma concentration[41], as well as barley beta-glucan consumption, which also increases PYY plasma concentration and, in addition, reduces ghrelin plasma concentration [42]. The increase in PYY plasma concentration may be due to a direct effect of short chain fatty acids (SCFA), produced from the microbial fermentation in the large intestine, on the GPR43 receptor present in the L cells of the intestinal mucosa, which are responsible for the secretion of PYY [37, 43]. The absence of significant differences in plasma concentrations of these hormones observed in this study suggests that the dose of beta-glucan tested may have been insufficient to increase the production of SCFA in dogs. Furthermore, the absence of differences between fecal pH values ​​of the two experimental groups may be indicative that the fermentative processes occurred similarly in the GIT of animals. We suggest studies evaluating the supplementation of diets for dogs with beta-glucans include the analysis of SCFA concentrations in feces to determine the potential of these compounds in influencing the microbial fermentation. In addition, significant differences in plasma concentrations of PYY and ghrelin were not observed either in dogs that received diets composed of highly fermentable fibers or poorly fermentable fibers [19]. The authors of that study also question whether the contrast between the two diets evaluated was sufficient to generate differences related to the plasma concentrations of these hormones.

The beta-glucan consumption alone did not influence the complete blood counts of dogs. However, there was an increase in the number of red blood cells, hematocrit percentage and hemoglobin concentration seven days after the second dose of vaccine. Studies in rats and humans indicate that the consumption of diets supplemented with fermentable fibers results in an increased production of folic acid by the intestinal microbiota [44, 45], being this vitamin essential for the maturation of red blood cells [46]. In addition, some studies in rats show that supplementation of diets with fermentable fibers results in an increase in intestinal iron absorption [47, 48], which is an important mineral for the synthesis of hemoglobin [49]. This effect can be explained by the reduction of intestinal pH due to microbial fermentation, which promotes the conversion of ferric ions to ferrous ions, as well as the proliferation of epithelial cells, consequently increasing the intestinal absorption surface [50]. However, fecal pH was not influenced by dietary supplementation with beta-glucan in the present study, which indicates that fermentative processes probably occurred in a similar way in the animals of the two experimental groups. Therefore, more studies are needed to elucidate the relationship between the presence of fermentable fibers in the GIT and the alteration of hematological parameters in dogs.

Some studies have also demonstrated the influence of beta-glucans on the immune system [12, 51–53]. Hence, vaccination of animals was performed in order to establish a form of challenge for the immune response.

In the present study, there was no influence of beta-glucan supplementation on the white blood cell count (WBC) of vaccinated animals. Similarly, supplementation of fish diet with beta-(1,3),(1,6)-glucan did not influence the total WBC of vaccinated animals compared with animals that did not receive supplementation [54]. In swine, dietary supplementation with beta-(1,3),(1,6)-glucan resulted in increased *in vitro* lymphocyte proliferation 14 days after vaccination [55]. An increase in lymphocyte concentration was also observed in an *in vivo* study with pigs fed a diet supplemented with beta-(1,3),(1,6)-glucan and challenged with *Escherichia coli* lipopolysaccharide [56]. However, it is important to note that in the three studies mentioned, a beta-glucan structurally different (beta-(1,3)-(1,6); branched structure) from the oat beta-glucan (beta-(1,3)-(1,4); non-branched structure) was used [1]. These structural differences may be related to distinct patterns of immune response modulation. It is known that the presence of branching in the beta-glucan structure enhances the recognition of this compound by dectin-1, the major receptor related to beta-glucan recognition [57]. However, excessive amount of branching may impair the interactions between beta-glucans and their receptors since compounds formed by non-branched chains showed an increase in their *in vitro* stimulatory capacity as their concentrations increased [58]. In fact, it is noted that the results related to WBC may vary according to the origin of the beta-glucan used and the species used in the study. Furthermore, in this study, all experimental animals showed total leukocyte, neutrophil, lymphocyte, monocyte and eosinophil counts within the reference values ​​for dogs. These results demonstrate the absence of a strong immune response as a result of dietary supplementation with oat beta-glucan extract.

The pattern observed in IL-4 serum concentrations in animals of the present study can be explained by the fact that vaccine was administered subcutaneously. The administration of antigens by this route can form persistent precipitates at the site of application, which dissolve relatively slowly [59]. This slow release of antigens may result in a process of chronic activation of T lymphocytes, leading to increased production of IL-4. More specifically, in normal situations, lymphocytes produce small amounts of IL-4 at the onset of cellular activation. However, as the stimulus persists, production of this cytokine increases until the threshold for a Th2 response polarization is reached and IL-4 production consequently increases. In addition, the predominance of a Th2 response inhibits the development of a Th1 response, which is characterized by a higher concentration of INFɣ [60]. Probably, this is the reason why it was not possible to detect significant amounts of INFɣ by the immunoenzymatic assay performed in the present study.

However, it was observed a reduction in IL-4 serum concentrations of animals fed the beta-glucan supplemented diet seven days after the first dose of vaccine. This result suggests that the beta-glucan extract used is capable of inhibiting the development of a Th2 response. Although differences in the detectable amounts of INFɣ and WBC were not observed, results related to IL-4 serum concentrations may be positive considering the type of vaccine administered to animals.

In general, according to the results observed in the present study, it is suggested that the use of oat beta-glucan extract as a dietary supplement has beneficial effects for dogs, mainly due to the reduction of blood concentrations of total cholesterol, LDL-c and VLDL-c and probably stimulating the immune response of vaccinated dogs. However, since this is an initial evaluation, further studies are needed to verify the effects of higher doses of beta-glucan in order to establish the relationship between oat beta-glucan and animal health.

## Conclusion

It is concluded that oat beta-glucan extract can be used as a dietary supplement for dogs at a dose of 10 g/kg of food, being effective in reducing blood concentrations of total cholesterol, LDL-c and VLDL-c as well as CTTAD of nutrients. These results demonstrate the potential of using this type of beta-glucan in the diet of obese animals. In addition, by reducing the predominance of a Th2 response, oat beta-glucan can positively modulate the vaccine response of animals.
